# Expression of Lymphatic Markers in the Adult Rat Spinal Cord

**DOI:** 10.3389/fncel.2016.00023

**Published:** 2016-02-09

**Authors:** Alexandra Kaser-Eichberger, Falk Schroedl, Lara Bieler, Andrea Trost, Barbara Bogner, Christian Runge, Herbert Tempfer, Pia Zaunmair, Christina Kreutzer, Andreas Traweger, Herbert A. Reitsamer, Sebastien Couillard-Despres

**Affiliations:** ^1^University Clinic of Ophthalmology and Optometry, Research Program for Experimental Ophthalmology and Glaucoma Research, Paracelsus Medical UniversitySalzburg, Austria; ^2^Institute of Anatomy, Paracelsus Medical UniversitySalzburg, Austria; ^3^Institute of Experimental Neuroregeneration, Paracelsus Medical UniversitySalzburg, Austria; ^4^Spinal Cord Injury and Tissue Regeneration Center Salzburg, (SCI-TReCS), Paracelsus Medical UniversitySalzburg, Austria; ^5^Institute of Tendon and Bone Regeneration, Paracelsus Medical UniversitySalzburg, Austria; ^6^Austrian Cluster for Tissue RegenerationVienna, Austria

**Keywords:** spinal cord, macrophage, PROX1, LYVE-1, Iba1, OLIG2, lymphatics, spinal cord injury

## Abstract

Under physiological conditions, lymphatic vessels are thought to be absent from the central nervous system (CNS), although they are widely distributed within the rest of the body. Recent work in the eye, i.e., another organ regarded as alymphatic, revealed numerous cells expressing lymphatic markers. As the latter can be involved in the response to pathological conditions, we addressed the presence of cells expressing lymphatic markers within the spinal cord by immunohistochemistry. Spinal cord of young adult Fisher rats was scrutinized for the co-expression of the lymphatic markers PROX1 and LYVE-1 with the cell type markers Iba1, CD68, PGP9.5, OLIG2. Rat skin served as positive control for the lymphatic markers. PROX1-immunoreactivity was detected in many nuclei throughout the spinal cord white and gray matter. These nuclei showed no association with LYVE-1. Expression of LYVE-1 could only be detected in cells at the spinal cord surface and in cells closely associated with blood vessels. These cells were found to co-express Iba1, a macrophage and microglia marker. Further, double labeling experiments using CD68, another marker found in microglia and macrophages, also displayed co-localization in the Iba1+ cells located at the spinal cord surface and those apposed to blood vessels. On the other hand, PROX1-expressing cells found in the parenchyma were lacking Iba1 or PGP9.5, but a significant fraction of those cells showed co-expression of the oligodendrocyte lineage marker OLIG2. Intriguingly, following spinal cord injury, LYVE-1-expressing cells assembled and reorganized into putative pre-vessel structures. As expected, the rat skin used as positive controls revealed classical lymphatic vessels, displaying PROX1+ nuclei surrounded by LYVE-1-immunoreactivity. Classical lymphatics were not detected in adult rat spinal cord. Nevertheless, numerous cells expressing either LYVE-1 or PROX1 were identified. Based on their localization and overlapping expression with Iba1, the LYVE-1+ cell population likely represents a macrophage subpopulation, while a significant fraction of PROX1+ cells belong to the oligodendrocytic lineage based on their distribution and the expression of OLIG2. The response of these LYVE-1+ and PROX1+ cell subpopulations to pathological conditions, especially in spinal cord inflammatory conditions, needs to be further elucidated.

## Introduction

Two crucial roles are carried out by the lymphatic system: (1) fluid homeostasis via the drainage of extracellular fluid; and (2) immune defense through the transport of antigens and immune cells to the lymph nodes (Alitalo, 2011; Card et al., 2014). To fulfill these functions, the lymphatic system is widespread throughout the human body (Breslin, 2014). Nevertheless, a few tissues, such as the (inner) eye (Streilein, 2003) and the central nervous system (CNS; Ransohoff and Engelhardt, 2012), lack a “classical” lymphatic system and are therefore considered to be alymphatic. The epithet “classical” refers to the presence of vessels with a lymphatic phenotype, i.e., with endothelial cells expressing the surface receptors VEGFR3 or LYVE-1 and membrane bound components, such as podoplanin, or transcription- factors, such as PROX1 or FOXC2 (Banerji et al., 1999; Jackson et al., 2001; Sleeman et al., 2001; Jackson, 2007).

Over the last decades, research of the lymphatic system underwent a tremendous boost based on the introduction of these aforementioned markers. While identification of lymphatics within a tissue remains challenging due to the small caliber of the vessels and their structural similarity with small-caliber blood vessels, the markers introduced now allow reliable identification (Sleeman et al., 2001). The crux with lymphatic markers however is that no exclusive marker has been identified so far (Sleeman et al., 2001) since most lymphatic markers are also expressed on cells other than lymphatic endothelium (Matsui et al., 1999; Schroedl et al., 2008). Therefore, for the unequivocal identification of lymphatics, there is a consensus that a combination of several markers is necessary, as recently stated for the inner eye (Schroedl et al., 2014) or under pathological conditions (Van der Auwera et al., 2006). Hence, we chose for this study the combination of the transcription factor PROX1, a homeo-box protein that retains its activity in nuclei of lymphatic endothelium in adulthood (Wigle and Oliver, 1999; Wilting et al., 2002) and LYVE-1, a membrane-bound glycoprotein and one of the best characterized markers of lymphatic endothelium (Jackson, 2004; Baluk and McDonald, 2008).

Being part of the CNS, which is considered to be alymphatic, the existence of structures or cells expressing lymphatic markers has not been thoroughly investigated within the spinal cord. The characterization of these putative lymphatic cells is particularly relevant for repair mechanisms considering that lymphatic cells can organize into a lymphatic system during pathological processes (Paavonen et al., 2000; Kerjaschki et al., 2004; Maruyama et al., 2005; Zumsteg et al., 2009; Tammela and Alitalo, 2010; Kerjaschki, 2014; Tempfer et al., 2015).

In this study, we scrutinize with histological methods the presence of potential lymphatic system or cells expressing lymphatic markers in the healthy, as well as the injured, adult rat spinal cord.

## Materials and Methods

### Specimens

All experiments were performed in conformity with the Directive (2010/63/EU) of the European Parliament and of the Council and were approved by the national animal health commission (Land Salzburg, Referat Gesundheitsrecht und Gesundheitsplanung Referat 9/01). Female Fisher 344 rats of approximately 3 months of age were used for this study. For histological analysis, rats received an overdose of anesthetics (ketamine/xylazine/ acepromazine; i.p.) prior to transcardial perfusion with NaCl 0.9% followed by phosphate buffered saline (PBS) containing 4% formaldehyde. The spinal cord and skin samples from the thigh were dissected, further fixed by immersion (1 h, room temperature, RT) and rinsed in PBS. Spinal cord and skin samples were transferred into PBS containing 15% sucrose (12 h at 4°C), embedded in tissue embedding medium (Slee Technik, Mainz, Germany) and frozen using liquid nitrogen-cooled methylbutane and stored at −20°C until further processing.

### Spinal Cord Contusion Injury (SCI)

Rats were anesthetized using 1.6% Isoflurane-oxygen mix. For analgesia, 0.03 mg/kg bodyweight (bw) Buprenorphine (Bupaq^®^, 0.3 mg/mL, Richterpharma, Wels, Austria) was injected sub-cutaneous (SC) 30 min prior to surgery. To prevent hypothermia, body temperature was maintained by a rectal sensor-coupled to a heating pad. Heart frequence and oxygen saturation was monitored throughout the whole surgical procedures (SomnoSuite^®^, KENT). During surgery, the dorsal aspect of the vertebra at thoracic level 8 was removed to expose the dura mater and the spinal cord. Contusion was performed using an Infinite Horizon impactor (IH-Impactor^®^, Precision Systems and Instrumentation, LLC with a force of 200 kdyn with immediate withdrawal). After that the absence of bleeding has been confirmed, paravertebral muscles were sutured and the skin was closed using a skin stapler. To prevent pain and infections after surgery, 1–2 mg/kg bw Meloxicam (Metacam^®^ 5 mg/ml) and 10 mg/kg bw Enrofloxacin (Baytril^®^ 25 mg/ml) was injected SC daily for 5 days. Additionally, 0.01 mg/kg bw Buprenorphine injected SC twice a day for 2 days post surgery. As bladder function was impaired after SCI, bladder was manually voided 2–3 times per day. Perfusion for histological analysis was performed 14 days post-lesion.

### Immunohistochemistry

Spinal cord and skin samples were sectioned with a cryostat (HM 550, Microm, Walldorf, Germany) in serial sections of 16 μm, collected on adhesion slides (Superfrost Plus; Thermo Scientific, Wien Austria) and air-dried for 1 h at RT. Sections were rinsed 5 min in Tris-buffered saline (TBS; Roth, Karlsruhe, Germany) and incubated for 1 h at RT in TBS containing 5% donkey serum (Sigma-Aldrich, Wien, Austria), 1% bovine serum albumin (BSA; Sigma-Aldrich), and 0.5% Triton X-100 (Merck, Darmstadt, Germany). Sections were then rinsed for 5 min in TBS and further incubated for single and double immunohistochemistry with the primary antibodies (Table 1) diluted in TBS, containing 1% BSA and 0.5% Triton X-100, for 12 h at RT. Sections were rinsed four times for 5 min in TBS and the primary antibodies visualized by corresponding Alexa488-, or Alexa555-conjugated antibodies (Invitrogen, Karlsruhe, Germany) diluted 1:1000 in TBS, containing 1% BSA and 0.5% Triton X-100 (1 h at RT). Subsequently, the sections were rinsed 5 min in TBS followed by a 10 min nuclear labeling with 4′,6-Diamidino-2 phenylindole dihydrochloride (DAPI, 0.25 μg/mL; VWR, Vienna, Austria). Finally, sections were rinsed three times for 5 min in PBS and embedded in TBS-glycerol (1:1 at pH 8.6).

**Table 1:** **Primary antibodies used in this study**.

Markers	Symbols	Hosts	Company	Dilution
Prospero homeobox protein 1	PROX1	Mouse	Acris; Herford, Germany	1:500
Lymphatic vessel endothelial hyaluronan receptor 1	LYVE-1	Rabbit	Acris; Herford, Germany	1:50
Cluster of differentiation 68	CD68	Rabbit	Abcam; Cambridge, UK	1:500
Ionized calcium-binding adapter molecule 1	Iba1	Goat	Wako Chemicals; Neuss, Germany	1:500
Protein-gene product 9.5	PGP9.5	Guinea pig	Merck Millipore; Vienna, Austria	1:500
Oligodendrocyte transcription factor 2	OLIG2	Rabbit	Merck Millipore; Vienna, Austria	1:300
Major histocompatibility complex class II	MHCII	Mouse	Abcam; Cambridge, UK	1:250

Rat skin sections were used to validate the LYVE-1 and PROX1 primary antibodies. Additionally, to avoid possible cross-reactivity in experiments involving two or more primary antibodies, successive incubations have been performed (i.e., primary and secondary antibodies for epitope one, followed by incubation with primary and secondary antibodies for epitope two). Negative controls were performed by omission of the primary antibodies during incubation and resulted in absence of immunoreactivity.

### Documentation

Micrographs of immunohistostainings were acquired using a confocal laser scanning unit (Axio ObserverZ1 attached to LSM710, Zeiss, Göttingen, Germany; ×20 dry or ×40 and ×60 oil immersion objektive lenses, with numeric apertures 0.8, 1.30, and 1.4, respectively; Zeiss). All images presented here consist of confocal images in single optical section mode. Negative controls were recorded with identical laser settings as used for documentation of corresponding primary antibodies.

## Results

**Figure 1:**
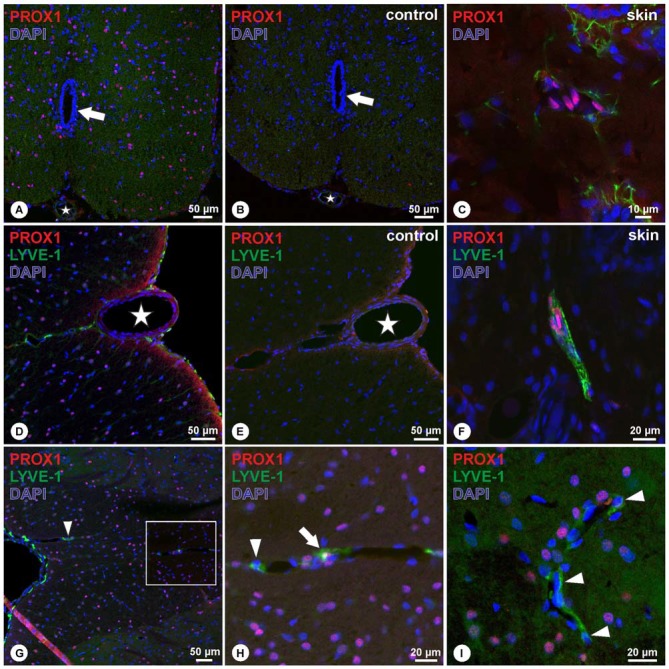
**(A–C)** In cross sections of the spinal cord, PROX1-immunoreactivity (red) was detected in numerous nuclei (DAPI, blue) throughout the gray and white matter without preference, as indicated by purple mixed color **(A)**, whereas negative controls revealed absence of immunoreactivity **(B)**. Asterisk **(A,B)** indicates ventral spinal artery, arrow **(A,B)** indicates the central canal. Controls in cross sections of rat skin **(C)** revealed nuclear Prox1 immunoreactivity as in **(A)**. **(D–F)** Double-immunohistochemistry of PROX1 (red) and LYVE-1 (green) reveals LYVE-1+ cells lacking PROX1-immunoreactivity on the surface of the spinal cord and following the anterior sulcal arteries **(D)**. Immunoreactivity was absent in negative controls **(E)**. Asterisk in **(D,E)** indicates ventral spinal artery. Skin controls revealed PROX1+ nuclei surrounded by LYVE-1-immunoreactivity **(F)**. Blue: DAPI. **(G–I)** Double-immunohistochemistry of PROX1 (red) and LYVE-1 (green) reveals LYVE-1+ cells on the surface of the posterior spinal cord lacking PROX1-immunoreactivity **(G)**, following the posterior spinal arteries (arrowhead). While occasionally cells were detected with apparent association of PROX1 and LYVE-1 (arrow in **H**), the majority of cells displayed immunoreactivity for LYVE-1 only (arrowheads in **H,I**). **(H)** represents magnification of the boxed area in **(G)**. Blue: DAPI.

In the rat adult spinal cord, PROX1-immunoreactivity was detected in numerous nuclei evenly distributed throughout the gray and white matter. Nuclei of the central canal lacked PROX1-immunoreactivity (Figure 1A). Absence of immunoreactivity was observed in the negative controls (Figure 1B), whereas in rat skin, used as positive control, PROX1-positive nuclei were bordering luminal structures (Figure 1C). Detection of the lymphatic endothelial marker LYVE-1 revealed numerous LYVE-1-expressing cells on the surface of the spinal cord, surrounding the ventral spinal artery, and following the sulcal arteries into the anterior median sulcus (Figure 1D). Double labeling experiments demonstrated that cells expressing LYVE-1 did not possess PROX1-positive nuclei (Figure 1D). Corresponding negative controls were lacking immunoreactivity (Figure 1E), whereas controls in rat skin showed cells with PROX-1 positive nuclei surrounded by LYVE-1 immunoreactivity (Figure 1F). Identical results were obtained on the dorsal side of the spinal cord, i.e., LYVE-1-immunoreactive cells were detected on the spinal cord surface and also in association with vessels of the dorsal spinal arteries entering the parenchyma (Figures 1G,H). Occasionally, PROX1-immunoreactive nuclei were in close vicinity with LYVE-1+ structures, which likely reflected expression of single markers in adjacent cells (Figure 1H). Nevertheless, the overwhelming majority of LYVE-1-positive cells surrounding vessels lacked PROX1 immunoreactivity (Figure 1I).

**Figure 2:**
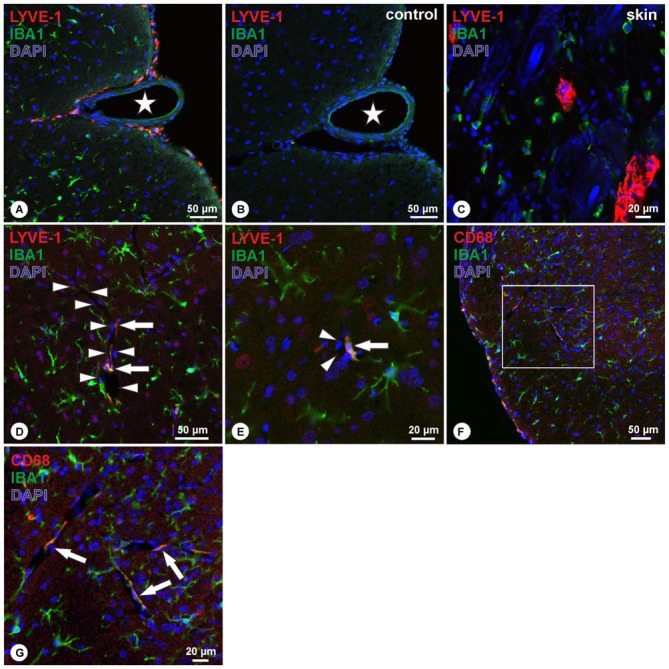
**(A–C)** Double-immunohistochemistry of LYVE-1 (red) and Iba1 (green) reveals LYVE-1+/Iba1+ cells on the surface of the spinal cord, following the anterior sulcal arteries **(A)**, whereas negative controls revealed absence of immunoreactivity **(B)**. Asterisk in **(A,B)** indicates ventral spinal artery. Skin controls revealed DAPI+ nuclei (blue) surrounded by LYVE-1-immunoractivity **(C)**, and dispersed cells showing immunoreactivity for Iba1 only. **(D,E)** Within the spinal cord parenchyma, double immunohistochemistry for LYVE-1 (red) and Iba1 (green) reveals cells co-localizing for both markers (arrows in **D,E**) bordering spinal cord blood vessels (arrowheads in **D,E**), whereas the majority of cells displayed immunoreactivity for Iba1 only. Blue: DAPI. **(F,G)** Double-immunohistochemistry of CD68 (red) and Iba1 (green) reveals CD68+/Iba1+ cells on the surface of the spinal cord **(F)**, and on cells bordering spinal cord blood vessels (arrows in **G**). **(G)** Higher magnification of boxed area in **(F)**. Blue: DAPI.

Double immunohistochemistry with LYVE-1 and Iba1 revealed a co-localization of both markers in cells located at the spinal cord surface (Figure 2A). Within the spinal cord, LYVE-1+/Iba1+ cells surrounding blood vessels were detected, whereas cells within the spinal cord parenchyma solely displayed immunoreactivity for Iba1 (Figures 2D,E). Cells displaying immunoreactivity exclusively for LYVE-1 were not observed. Double immunohistochemistry for CD68 and Iba1 revealed an identical pattern, i.e., CD68+/Iba1+ cells were detected on the spinal cord surface (Figure 2F), following the spinal cord arteries (Figures 2F,G). Cells immunoreactive exclusively for CD68 were not observed. Corresponding negative controls ascertained the absence of immunoreactivity (Figure 2B). In contrast, in the rat skin positive controls, two distinct cell populations were observed expressing either LYVE-1 or Iba1 without apparent co-localization (Figure 2C).

**Figure 3:**
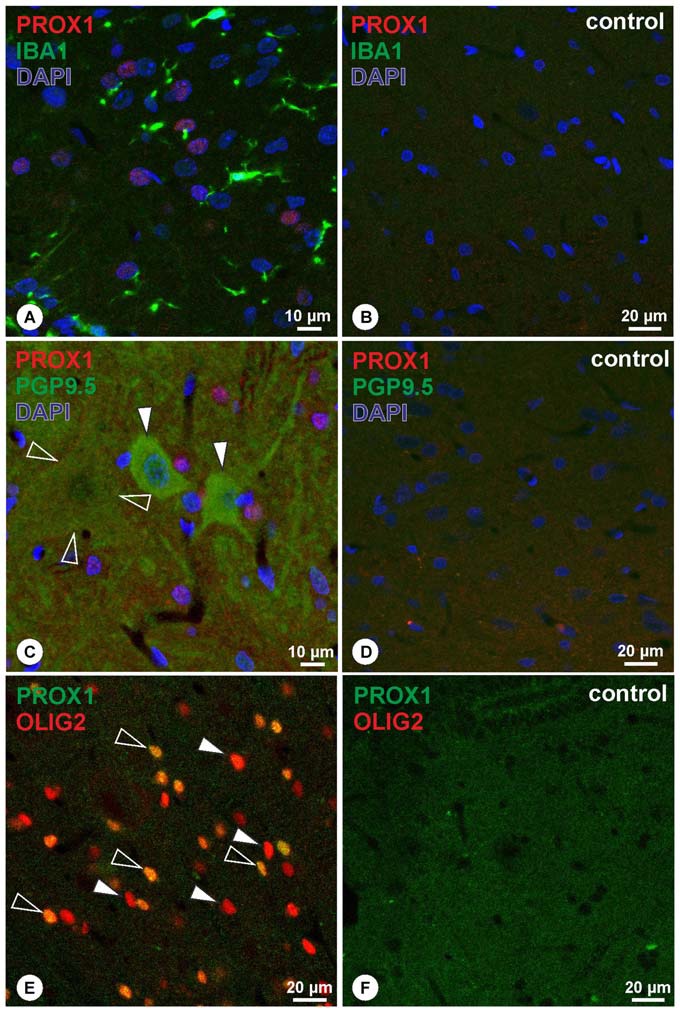
**(A,B)** Double-immunohistochemistry of PROX1 (red) and Iba1 (green) reveals that PROX1 positive nuclei (purple mixed color) are not associated with Iba1-positive cells **(A)**, whereas negative controls lack immunoreactivity **(B)**. Blue: DAPI. **(C,D)** Double-immunohistochemistry of PROX1 (red) and the pan-neuronal marker PGP9.5 (green) reveals that PROX1-positive nuclei (purple mixed color) are not associated with neurons of the spinal cord (**C**; arrowheads point to small neurons, open arrowheads outline a faint immunoreactive large neuron), whereas negative controls lack immunoreactivity **(D)**. Blue: DAPI. **(E,F)** Double-immunohistochemistry of PROX1 (green) and OLIG2 (red) reveals co-localized nuclei (**E**; yellow mixed color, open arrowheads), whereas some nuclei display OLIG2-immunoreactivity only (**E**; arrowheads). Immunoreactivity was absent in corresponding negative controls **(F)**.

To further characterize the PROX1+ cells within the spinal cord, PROX1-immunohistochemistry was combined with either Iba1, the pan-neuronal marker PGP9.5, or the oligodendrocyte lineage marker OLIG2. These double labeling experiments revealed that PROX1+ nuclei were not associated with Iba1 (Figure 3A), while corresponding controls showed absence of immunoreactivity (Figure 3B). Similarly, PGP9.5-immunoreactivity was not associated with PROX1+ nuclei in both small- and large-sized neurons (Figure 3C). Corresponding negative controls were not immunoreactive (Figure 3D). In contrast, the combination of PROX1 and OLIG2 revealed extensive co-localization of both markers in nuclei throughout the spinal cord (Figure 3E). Characterization in five randomly chosen micrographs of approximately 200 nuclei expressing either marker revealed that 46% were PROX1+/OLIG2+, whereas 42% showed immunoreactivity for OLIG2 only and 12% displayed immunoreactivity for PROX1 only. PROX1/OLIG2 immunoreactivity was absent in corresponding negative controls (Figure 3F).

**Figure 4:**
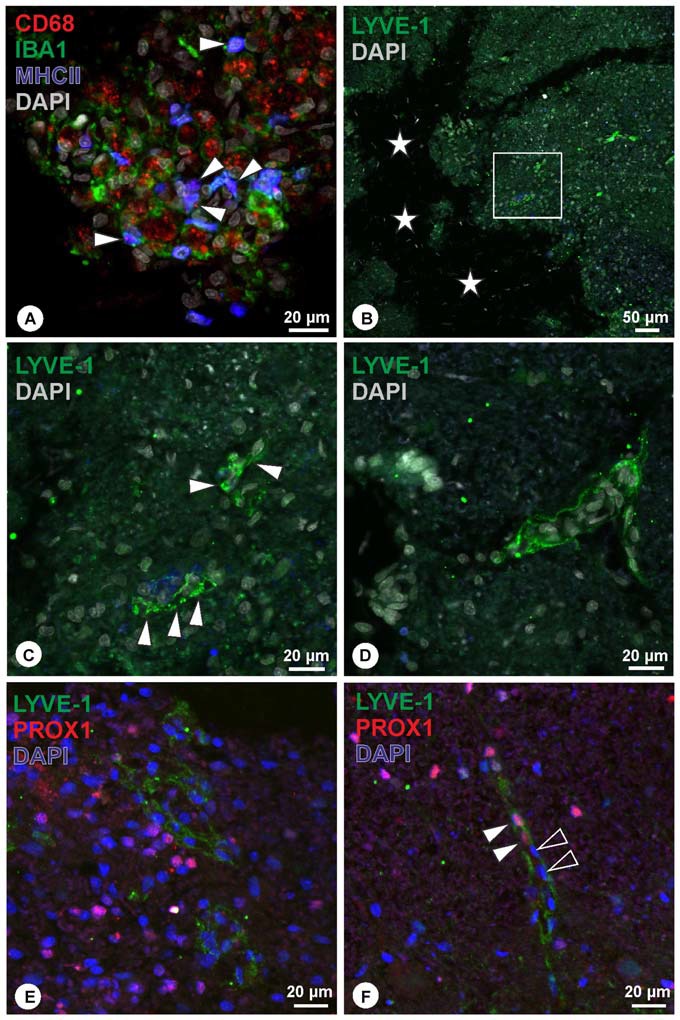
**(A)** Within the lesion site, many cells were detected displaying immunoreactivity for CD68 (red), a subpopulation of which co-localized for MHCII (blue, arrowheads), while Iba1-immunoreactive cells (green) were intermingling. **(B,C)** Close to the lesion center (asterisks), organized LYVE-1 immunoreactive structures (green) were detected (**C**, arrowheads; magnification of boxed area in **B**), displaying vessel-like appearance. DAPI: gray. **(D)** Another example of vessel-like structure expressing LYVE-1. DAPI: gray. **(E,F)** Double immunohistochemistry of LYVE-1 (green) and PROX1 (red) revealed that the majority of LYVE-1 immunoreactive structures were not associated with PROX1-positive nuclei **(E)**. In few instances, PROX1 positive nuclei were detected closely associated with LYVE-1 immunoreactive structures (**F**, arrowheads), while other nuclei in proximity were lacking PROX1 (**F**, open arrowheads).

Preliminary investigation in lesioned spinal cord revealed cell debris and cells accumulation within the lesion site. Many cells displayed immunoreactivity for CD68 (Figure 4A), while a subpopulation of CD68+ cells was also immunoreactive for MCHII (Figure 4A). Iba1-immunoreactive cells were interspersed between CD68+/MHCII+ cells (Figure 4A). The amount of CD68-immunoreactive cells decreases towards the periphery of the lesion site, and similarly Iba1+ cells were less frequently detected. Within the lesion site, LYVE-1+ cells assembled in structures that were not observed in the unlesioned tissue (Figures 4B,C). These LYVE-1+ structures appeared organized (Figure 4C), where associated with elongated nuclei, and displayed vessel-like formation (Figures 4C,D). Double immunohistochemistry with LYVE-1 and PROX1 revealed that the majority of LYVE-1 immunoreactive structures were not associated with PROX1-poitive nuclei (Figure 4E). However, in few instances, PROX1-positive nuclei were closely related to LYVE-1 immunoreactive structures (Figure 4F).

## Discussion

The dominant opinion in current textbooks considers the CNS as an alymphatic environment (Iliff and Nedergaard, 2013). However, with the availability of lymphatic markers, this dogma has been recently revisited and challenged (Aspelund et al., 2015; Louveau et al., 2015; Wood, 2015). Therefore, we investigated adult rat spinal cord for the expression of components specific for lymphatic endothelium, namely the membrane bound glycoprotein LYVE-1 in combination with the transcription factor PROX1. LYVE-1 as a marker for lymphatic endothelium was first identified in 1999 by Banerji et al. (1999). However, its expression is not exclusive for lymphatics, since it is also detected among others on liver sinusoids (Mouta Carreira et al., 2001), pulmonary (Favre et al., 2003) and renal glomerular capillaries (Lee et al., 2011) and its expression pattern may also change along the lymphatic vascular tree (Baluk and McDonald, 2008).

The function of LYVE-1 in cells of these various systems is still not fully understood (Jackson, 2009). On the other side, PROX1 is a homeobox transcription factor and a key player in the development of many organ systems, such as the enterohepatic system or heart (Oliver et al., 1993; Sosa-Pineda et al., 2000). In the CNS, PROX1 is known as a critical regulator of neurogenesis and neuronal differentiation (reviewed in Stergiopoulos et al., 2014). In nuclei of lymphatic endothelial cells, PROX1 expression persists into adulthood in physiological as well as in pathological conditions (Wilting et al., 2002). Here, PROX1 is considered to be a master gene (Hong and Detmar, 2003) in lymphatic endothelial progenitor cells controlling the expression of other lymphatic markers (for review, see Yang and Oliver, 2014).

Although we could readily detect these two markers closely associated in lymphatic endothelial cells of the skin, our lymphatic control tissue, our study demonstrated the absence of PROX1+/LYVE-1+ cells in the spinal cord. On the other hand, we detected numerous cells expressing either of these markers, however, under physiological conditions, these were not associated with lymphatic vessel-like structures. The LYVE-1+ cells were found at the surface of the spinal cord or closely located to spinal cord blood vessels. These LYVE+ cells co-localized with the macrophage and microglia markers Iba1, and most likely also CD68, as observed by the almost overlap of the latter with Iba1-immunoreactivity. This marker combination, together with the localization in vicinity to blood vessels highly suggests that this cell population represents macrophages.

Indeed, brain macrophages are mainly situated close to cerebral blood vessels under physiological conditions (Bogie et al., 2014) and LYVE-1 expression in macrophages is well established in other systems (Schledzewski et al., 2006; Cho et al., 2007; Schroedl et al., 2008). While macrophages play an important role during pathological processes (Shechter and Schwartz, 2013), the role of the LYVE-1+ macrophage sub-population in the spinal cord in physiological and pathological conditions remains to be elucidated. Following spinal cord contusion injury, we could detect a re-organization and redistribution of cells expressing LYVE-1 within the lesioned parenchyma. Some of these cells reorganized in structures resembling putative pre-vessels (Figure 4). Therefore, following lesion, there is a solid evidence to reconsider the possibility of lymphatics within the spinal cord, and most likely also within the brain.

PROX1+ cells were found widespread throughout the spinal cord parenchyma. These PROX1-positive nuclei were neither detected in association with the microglia/macrophage marker Iba1, nor with the pan-neuronal marker PGP9.5, thus ruling out that they represented microglia or neurons, respectively. Instead, PROX1+ nuclei were co-localized with the oligodendrocyte lineage marker OLIG2. The co-existence of these two markers within a cell population is particularly puzzling since Olig2 and PROX1 have been reported to reciprocally suppress the expression of each other during development and in forced-expression experiments (Kaltezioti et al., 2014; Stergiopoulos et al., 2014). Nevertheless, in a study addressing the gene expression pattern found in the various cell populations of the CNS, Cahoy et al. (2008) found that Olig2 and PROX1 are enriched in the oligodendroglial lineage. Moreover, the expression of PROX1 was five times higher in myelin-producing oligodendrocytes as compared to oligodendrocyte precursor cells (OPCs). Hence, the PROX1+/Olig2+ cell population detected in the spinal cord probably consists of mature oligodendrocytes. Whether the small percentage of cells expressing solely PROX1 constitutes a distinct population, or a population with low Olig2 expression, remains to be deciphered.

With this study, we confirmed the assumption that classical lymphatic vessels are absent from the healthy adult rat spinal cord. Nevertheless, recent data generated under pathological conditions suggested the development of lymphatic structures in otherwise alymphatic environments, as seen for example in cornea (Cursiefen et al., 2002) or tendon lesions (Tempfer et al., 2015). On the other hand, the existence of structures capable of fulfilling lymphatic vessel-like functions, as recently described for e.g., the Schlemm’s Canal of the eye (Aspelund et al., 2014), remains to be demonstrated in the spinal cord. The response of the LYVE-1 positive macrophages observed following spinal cord lesion needs to be further investigated as they may be the prerequisite for the formation of lymphatic-like structures (Maruyama et al., 2005; Alitalo, 2011; Ran and Montgomery, 2012; Kerjaschki, 2014).

## Author Contributions

FS, AK-E, SC-D, HAR conceived and designed the study and AK-E, FS, CK, LB, PZ performed the experiments or contributed to data acquisition. FS, AK-E, CK, LB, SC-D analyzed the data and AT, PZ, CK, HT, BB, CR contributed to data interpretation. AK-E, FS, SC-D wrote the manuscript and AK-E, FS, SC-D, HAR, HT, AT, PZ, LB, CK, AT, BB, CR critically reviewed the manuscript. All authors read and approved the final version of the manuscript.

## Conflict of Interest Statement

The authors declare that the research was conducted in the absence of any commercial or financial relationships that could be construed as a potential conflict of interest.
